# Anti-cyclic citrullinated peptide antibody is a good indicator for the diagnosis of rheumatoid arthritis

**DOI:** 10.12669/pjms.293.2924

**Published:** 2013

**Authors:** Asrul Abdul Wahab, Marlyn Mohammad, M. M Rahman, Mohd. Shahrir Mohamed Said

**Affiliations:** 1 Asrul Abdul Wahab, Department of Medical Microbiology & Immunology, Faculty of Medicine, The National University of Malaysia, Cheras 56000 Kuala Lumpur, Malaysia.; 2 Marlyn Mohammad, Department of Medical Microbiology & Immunology, Faculty of Medicine, The National University of Malaysia, Cheras 56000 Kuala Lumpur, Malaysia.; 3 M.M. Rahman, Department of Medical Microbiology & Immunology, Faculty of Medicine, The National University of Malaysia, Cheras 56000 Kuala Lumpur, Malaysia.; 4 Mohd. Shahrir Mohamed Said, Department of Internal Medicine, Faculty of Medicine, The National University of Malaysia, Cheras 56000 Kuala Lumpur, Malaysia.

**Keywords:** Rheumatoid arthritis, anti-CCP, Rheumatoid factor, Sensitivity, Specificity, Positive predictive value, Negative predictive value

## Abstract

***Objectives:*** Anti-cyclic citrullinated peptide (CCP) antibody has recently been used in the classification of rheumatoid arthritis (RA). This antibody is more specific than rheumatoid factor (RF) for the diagnosis of RA. The study objectives were to determine the sensitivity, specificity, positive and negative predictive values of anti-CCP in RA diagnosis.

***Methodology:*** Eighty RA patients and 80 non-RA individuals were included in the study. Blood was collected from both arms of study subjects and tested for anti-CCP and RF antibodies. Relevant clinical information and laboratory profiles of the RA patients were evaluated using patients’ medical records and Integrated Laboratory Management System (ILMS), respectively.

***Results*** ***:*** The sensitivity and specificity of anti-CCP were 35% and 100% respectively. The positive and negative predictive values were 100% and 61%, respectively. Positive anti-CCP was found significantly associated with multiple joint pain (p< 0.001) and hand’s joints pain (p=0.01), symmetrical joints involvement (p=0.015) and high CRP value (p<0.001). Anti-CCP was also found to have positive association with RF (p<0.001).

***Conclusion:*** Anti-CCP is highly specific for the diagnosis of RA. High positive predictive value should be taken into consideration for effective treatment.

## INTRODUCTION

Rheumatoid arthritis (RA) is a chronic systemic autoimmune disorder, which is primarily affecting the joints and may cause devastating complications. Genetic and environmental factors favour for the development of the disease. HLA class II molecules particularly HLA-DRB1 alleles are known to be present in RA patients.^1^ Recently, *Proteus mirabilis *has been implicated in the aetiopathogenetic mechanism of RA.^2^ Combination of smoking and HLA-DRB1 shared epitope alleles (SE) was shown to increase the risk of developing RA.^3^ RA is affecting about 1-2% of populations depending on the geographical distribution in the world. In Malaysia, the disease prevalence is 0.5%.^4^

Early diagnosis and treatment are important to avoid unwanted complications of the disease. Rheumatoid factor (RF) is used to help diagnosis of RA. In fact, according to previous criteria for diagnosis of RA provided by American College of Rheumatology in 1987, RF is the only laboratory criteria included. RF is sensitive but lack specificity. The sensitivity ranges between 25-95% while specificity ranges from 31-95%.^5^ High rates of false positive RF were found in other connective tissue diseases namely SLE and Sjogren’s syndrome and in patients with chronic hepatitis.^6^

Anti-cyclic citrullinated peptide (CCP) detection is one of the latest markers that have been introduced for the diagnosis of RA. The sensitivity of the assay is similar to RF but it has better specificity. The sensitivity ranges from 39-94% and the specificity ranges from 81-100%.^5^ Recently, the new criteria for diagnosis of RA have been introduced and anti-CCP together with RF is included in the criteria.^7^

This study was performed to determine the sensitivity, specificity, positive and negative predictive value in RA patients at UKM Medical Centre (UKMMC), Malaysia.

## METHODOLOGY

***Patients: ***The study was conducted from March 2010 until February 2011. Eighty rheumatoid arthritis (RA) patients were included in this study. All these patients were followed up under Rheumatology Unit UKMMC. The RA was considered based on the criteria provided by American College of Rheumatology 1987. Another 80 individuals without the disease were also recruited as negative controls group. Written consent to participate in the study was taken from all participants. Patients with idiopathic juvenile arthritis (IJA) were excluded from the study. About 5 ml of blood was acquired from all participants for anti-CCP and RF tests. Patients group’s medical records were looked through for demographic data as well as clinical signs and symptoms. Relevant laboratory investigations namely erythrocyte sedimentation rate (ESR) and C-reactive protein (CRP) were reviewed through the Integrated Laboratory Management System (ILMS) available in UKMMC laboratories.

***Anti-cyclic citrullinated peptide (CCP): ***The detection of anti-CCP was performed by ELISA, AESKULISA (Aesku, Wendelsheim, Germany). Standard protocol was followed according to manufacturer’s instruction. Results were reported qualitatively with reading of > 18U/ml was considered as positive.

***Rheumatoid factor (RF): ***In our laboratory, RF detection has been routinely tested by latex agglutination method as for the method mentions with the kit (VEDALAB, France). The result was reported qualitatively. Sera that agglutinate with the latex particle were considered as positive.

***Statistical analysis: ***The data was analyzed by chi-square using IBM SPSS software version 19.0. The p value of <0.05 was considered as significant. The sensitivity, specificity, positive and negative predictive value were calculated based on the standard table for sensitivity and specificity.

## RESULTS

Eighty patients were in included in this study. The age ranges from 20-86 years old in RA patients with mean age of 51 years. Female represents majority of the RA patients in this study (n=73). Majority of the patients were Malay ethnic group followed by Chinese and Indian. This is perhaps reflecting the demographic pattern of Malaysian population in which Malays are the main ethnic group followed by Chinese, Indian and other races. The data is shown in the Table-I.

**Table-I:** Demographic data of patients with rheumatoid arthritis (RA) and negative control

	*Rheumatoid Arthritis * *(n=80)*	*Negative Control (Non-Rheumatoid Arthritis)* *(n=80)*
Age (years)		
Range Mean	20-86 51	22-80 48
Gender		
Male Female	7 73	13 67
Race		
Malay Chinese India Others	45 24 9 2	43 23 14 -

Most of the patients (n=74, 93%) had complaint of joint pain over period of less than two years. Most of the time, the main presenting symptom was pain over bilateral small joints of the hands. Other clinical symptoms included pain over the knees and multiple joints pain. Forty-three percents (n=34) of the patients were RF positive. This group of patient was classified as seropositive RA. Thus, in the study majority of patients were seronegative RA.

**Fig.1:**
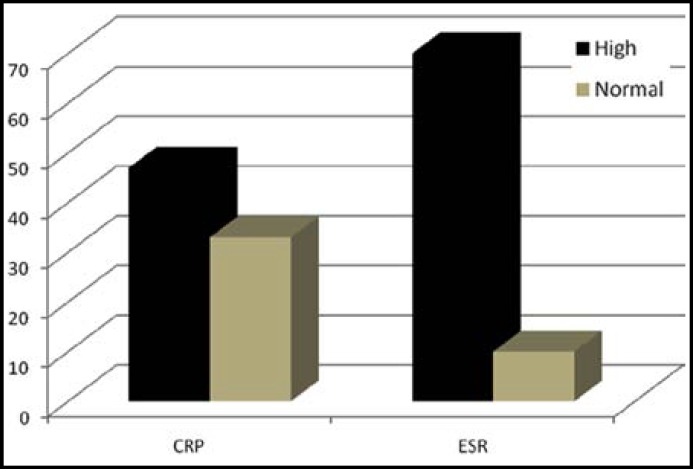
Bar chart showing number of RA patients with high and normal CRP and ESR levels, respectively

C-reactive protein and erythrocytes sedimentation rate (ESR) were then evaluated and the results are as shown in Fig.1. 47 and 70 patients had high CRP and ESR respectively. The value of > 0.5 mg/dL and >20 mm/hour are considered as raise in CRP and ESR respectively. CRP value ranges from 0.01 to 9.62 mg/dL with mean value of 1.26 mg/dL. ESR value ranges from 3 to 120 mm/hour with mean of 51.6 mm/hour. Bar chart showed the number of patients with high and normal CRP and ESR.

The sensitivity and specificity of anti-CCP were calculated at 35% and 100% respectively. The positive predictive value of anti-CCP is 100% and the negative predictive value is 61%. The sensitivity and specificity of RF on the other hand were calculated at 43% and 85% respectively. When both assays were combined, the sensitivity and specificity were 50% and 85% respectively. The number of positive and negative anti-CCP antibody tests is shown below (Table-II).

**Table-II:** Anti-CCP antibody results in RA and non-RA subjects

	*Rheumatoid Arthritis*	*Non-Rheumatoid Arthritis*	
Positive anti-CCP	28	0	28
Negative anti-CCP	52	80	132
Total	80	80	160

The positive anti-CCP is significantly associated with multiple joints involvement at presentation (p < 0.001) involvement of the hand’s joints (p=0.01), symmetrical joint pain (p=0.015), high CRP level (p < 0.001) and positive RF (p < 0.001). There were no significant correlation noted between positive anti-CCP and duration of illness as well as with high ESR. Table-III describes the clinical and laboratory parameters that have been analyzed in relation to anti-CCP antibody.

**Table-III:** Clinical presentation and Lab parameters

	*P value*
1. Joint involve	
Hands Knees Multiple joints 2. Duration ≤ 2 years > 2 years 3. Symmetrical involvement of the joint	0.01 0.132 <0.001 0.417 0.015
Laboratory parameters	
1. High CRP 2. High ESR 3. Positive Rheumatoid factor	<0.001 0.478 <0.001

## DISCUSSION

In this study, we found that the age of the patients with RA ranges from 20 to 86 years old with mean age of 51 years and female is the predominant gender. Anti-CCP antibody has 35% sensitivity, 100% specificity, 61% negative predictive value and 100% positive predictive value. We also found that, anti-CCP antibody is significantly associated with multiple joints pain, pain over the hands joint, symmetrical joint involvement, raise in CRP and rheumatoid factor (RF).

Studies conducted by several investigators showed the similar results as the current study in term of the disease is mainly affecting middle age group and female is the predominant gender.^8^^,^^9^ Local studies in Malaysia also exhibit the similar epidemiological features.^10^^,^^11^ Based on these findings, it is an established fact that rheumatoid arthritis commonly occurs in middle age adults and female is commonly affected among Malaysian patients.

It is widely acknowledged that bilateral joint pain or stiffness is among the most common complaint in rheumatoid arthritis patients, and majority will have pain involving multiple joint. In this study, most of the patients presented to the rheumatology clinic with bilateral pain or stiffness of small joints of the hands. According to the recently published classification criteria for rheumatoid arthritis, clinical synovitis is an important clinical presentation that should be looked into when evaluating rheumatoid arthritis patients.^7^ If the patient is having clinical synovitis irrespective of duration that clinically can be assessed by presence of joint swelling then the individual will be tested further. For previous diagnosis criteria of rheumatoid arthritis which was based on American College of Rheumatology criteria 1987, clinical presentation of joint pain, swelling and morning stiffness must be present for at least six weeks before diagnosis of rheumatoid arthritis can be considered. Multiple and symmetrical joint pains are the common presenting complaint of patients with rheumatoid arthritis.^12^

We found the sensitivity and specificity of anti-CCP were 35% and 100% respectively. The sensitivity and specificity of RF were 43% and 85% respectively. Interestingly, combination of both tests (either one of the test is positive) result in increase in sensitivity to 50% but reduction in specificity to 85%. Studies on anti-CCP have been conducted by many other researchers before, and it is noted that the performance is varied depending on the study population.^5^^,^^13^^,^^14^ Studies in Malaysia has also demonstrated different sensitivity of anti-CCP. One study showed sensitivity of 80.4% while in the other study the sensitivity is 66.7%.^10^^,^^11^ We postulate that, the sensitivity of anti-CCP is low in this study because majority of the patients belong to seronegative rheumatoid arthritis.

In this study we were more concerned with specificity of the anti-CCP. The result showed that anti-CCP has better specificity than RF in our cohort. In most of the previous studies, anti-CCP were shown to be far superior than RF in term of specificity.^15^ In our control group, RF was positive in patients with SLE, osteoarthritis and pregnant woman. Because of low specificity of RF, the diagnostic implication is lower. Thus, positive RF result must be interpreted with caution and other parameters like clinical presentation and inflammatory markers to be taken into consideration when making the diagnosis of RA. Interestingly, presence of either anti-CCP or RF will increase the sensitivity to 50%. Similar finding supported our finding where presence of either anti-CCP or RF increases testing sensitivity for diagnosis of rheumatoid arthritis.^15^ Interestingly, seropositivity for anti-CCP is associated with RF seropositivity among our study population consistent with several other works before.^11^^,^^16^ Thus, we think that the group of seronegative RA is probably encompasses of different clinical entity. Recent study showed that the seronegative RA is clearly has different genetic predisposition compared to seropositive group.^17^ In the latest Classification Criteria of Rheumatoid Arthritis 2010, both assays are included as laboratory markers to help diagnose the disease.^7^ The latest guideline highlights the importance of quantitative value of the marker. A higher value will give higher score. With incorporation of these laboratory markers within the diagnostic criteria, early RA cases will be identified. This will prompt treatment to be initiated as early as possible, and ensures prevention of complication of intractable joint and bone damage.

Strong correlation of anti-CCP with inflammatory markers particularly CRP has been well-demonstrated. Here we have demonstrated the significant association of anti-CCP sero positivity with raise in CRP. Our finding is consistent with previous studies which described raise in CRP occurred more common in anti-CCP positive than anti-CCP negative.^8^ Positive anti-CCP and high CRP level were shown to be the only significant predictor related to joint destructions.^18^ The strong correlation between positive anti-CCP and radiological evidence of joint damage in RA has been described. Extensive review also noted the risk for radiographic progression is greater in those with positive anti-CCP.^19^ However, in this study the use of anti-CCP as a prognostic marker is not evaluated thoroughly. Perhaps, another prospective study to quantitatively monitor level of anti-CCP with disease progression and treatment response will be desirable.

In conclusion, anti-CCP has a good specificity and positive predictive value in helping to establish diagnosis of RA. Further study is required to determine underlying genetic association in patients with RA in Malaysia and the prognostic value of anti-CCP in RA.

## Author contribution

AAW: Designed the study, performed laboratory tests (anti-CCP and RF), did data collection, statistical analysis and prepared the manuscript.

MM: Supervised the work, contributed in designing the study and editing the manuscript.

MMR: Did review and final approval of manuscript.

MSMS: Helped to recruit patients for this study.
